# In and out among the Hospitals

**Published:** 1888-05-19

**Authors:** 


### In and Out Among the Hospitals.

By a Secretary of Ten Years' Standing.

Copies of Annual Reports and Rules, Accounts of Meetings, Changes in the Staff, &c., should be addressed The Editor, The Lodge, Porchester Square, W.

### THE LONDON HOSPITAL.

The quinquennial appeal of governors of this hospital is being made this year, and has already met with the liberal response it deserves. The London is gradually becoming a free hospital, nearly 104,000 patients having been treated last year, three-fourths of whom received the benefits of the institution without any governor's letter or recommendation of any kind. Much care has been taken to ascertain the fitness of out-patients for relief, and a regular system of inquiry and inspection has been in use for some years, which, the report states, has been found to work satisfactorily. It had been urged that the dread of having inquiry made would deter many deserving cases from attending a second time; but last year the inquiries were carried further, and those cases that did not present themselves after the first visit were inquired about, the result proving that only two cases in 29 had been deterred from coming again. These were found deserving, and were requested to return as out-patients. The report, however, does not give any opinion as to whether deserving patients who have heard of the system abstain from applying for help altogether, but it is proved beyond dispute that hundreds of cases annually are rejected by the inspectors as unfit to receive relief from a charitable institution. The total income for the year, including £6,338 6*s.* 6*d.*, carried to account from the "five years' maintenance fund," was £57,789 8*s.* 11*d.*, and the expenditure, including £4,983 14*s.* 9*d.* for extensions and improvements, was £56,071 7*s.* 11*d.* £5,171 6*s.* was realised on Consols to meet a deficit of £6,684 0*s.* 6*d.*, standing over from 1886, and £500 was handed over to the medical college for extension and alterations, leaving a debit balance of £294 13*s.* 6*d.* at the end of the year. The House Governor's report shows that the cost per bed occupied has been £68 3*s.* 7*d.*, as against £68 13*s.* 5*d.* in the previous year. An accurate account has been kept of the cost of out-patients, and they are set down at 3*s.* 11¾*d.* per head; this is nearly 3*s.* more than the amount usually estimated as the cost of out-patients in metropolitan hospitals, but it tends in no small degree to keep down the cost of in-patients. The daily average number of beds occupied was 644, and the mean residence of each in-patient 28 days. Mr. A. H. Haggard resigned the post of secretary to the governors, and has been succeeded by Mr. G. Q. Roberts, M.A. Oxon. The seniority list of officers published in the annual report show that there have been no changes on the medical staff. For some reason the secretary's name is omitted from the list, though his clerk's appears, and this year the names of the instructor of anæsthetics, storekeeper, and surgery beadle have been added to the list of officers. The report is as usual accurately and carefully compiled, and following the example set by a West End hospital a ground plan and map showing the position of the hospital and the various railway routes by which it can be reached has been added.

### CHELTENHAM GENERAL HOSPITAL AND DISPENSARY.

The annual report of the Quarterly Board of the Cheltenham General Hospital and Dispensary gives some interesting details of the working of the principal medical charity in this favourite watering-place. The income of the year, including legacies (which at this hospital are carried to a separate account) was £5,288 12*s.* 7*d.*, and the expenditure £5,308 6*s.* 2*d.*, of which £1,221 9*s.* was spent on new buildings and structural improvements. Deducting this sum, the cost of the branch dispensary, and 1*s.* for each out-patient treated at the hospital, gives the cost per bed occupied at £61 17*s.* 6*d.* The branch dispensary appears to be an expensive adjunct to this charity; it cost last year £551 17*s.* 10*d.*, which was at the rate of 2*s.* 10½*d.* per out-patient. The total number of patients in the ward last year was 633, and the daily average number of beds occupied was 50¼. The out-patients treated at the hospital numbered 3,512, and those at the branch dispensary 3,860, The new buildings referred to above are a great improvement to the institution. They comprise new operating-room, accident ward, and mortuary. The lavatories, &c., have been virtually reconstructed, and the out-patients' department very much improved. The resources of the charity were sorely tried during the recent outbreak of typhoid fever, and every department of the hospital, especially the nursing branch, proved fully equal to the occasion. It is gratifying to find that the services then rendered are being recognised, the Mayor and Health Committee of the town having promised a contribution towards the extra expenses incurred by the hospital in the treatment of patients suffering under the epidemic. Mr. D. C. Leighton resigned the post of honorary secretary and treasurer, and at the annual general meeting a hearty vote of thanks was accorded to him, and he was elected a vice-president in recognition of the valuable services which he had rendered to the hospital. On his retirement the Board experienced great difficulty in providing a successor, and contemplated the necessity of appointing a salaried secretary, but in the emergency Colonel Croker King kindly volunteered his services, which the Board gladly accepted. Miss Oldham resigned the post of matron, and has been succeeded by Miss Tatham, of St. Thomas's Hospital. The Board did not let Miss Oldham leave them without placing on record their sense of the very efficient services she had rendered to the hospital during her two and a half years' service, especially by the improvement she effected in the nursing department, which, it is considered, owes its present efficiency to her skill and organization.

